# HIV-positive gay men’s knowledge and perceptions of Human Papillomavirus (HPV) and HPV vaccination: A qualitative study

**DOI:** 10.1371/journal.pone.0207953

**Published:** 2018-11-29

**Authors:** Daniel Grace, Mark Gaspar, Rachelle Paquette, Ron Rosenes, Ann N. Burchell, Troy Grennan, Irving E. Salit

**Affiliations:** 1 Dalla Lana School of Public Health, University of Toronto, Toronto, Canada; 2 Toronto General Hospital, Toronto, Canada; 3 Canadian HIV/AIDS Legal Network, Toronto, Canada; 4 St. Michael’s Hospital, Toronto, Canada; 5 British Columbia Centre for Disease Control, Vancouver, Canada; Moi University School of Medicine, KENYA

## Abstract

The human papillomavirus (HPV) is the most common sexually transmitted infection worldwide. Gay, bisexual, and other men who have sex with men (GBM) living with HIV are disproportionately impacted by HPV-associated anal cancer, with rates about 100-fold that of the general population. Fortunately, HPV vaccination has proven efficacy in preventing both anogenital warts (condyloma) in males and anal pre-cancers (anal intraepithelial neoplasia; AIN) in GBM up to the age of 26. We conducted in-depth, semi-structured interviews with 25 HIV-positive gay men in Toronto to gain an understanding of their knowledge and experiences related to HPV and the HPV vaccine. These participants were part of The HPV Screening and Vaccine Evaluation (HPV-SAVE) Study, and received invitations to have anal cancer screening from their primary care doctors. Interviews were analyzed following a Grounded Theory Approach. Most participants had not received the HPV vaccine. Men described a lack of prior knowledge of the health consequences of HPV for GBM living with HIV and financial barriers to vaccine access. Participants did not articulate concerns about vaccine safety. Men frequently reported initial beliefs that HPV was predominantly—or exclusively—a risk for females or young girls, and thus they had not considered the vaccine to be necessary. Some participants remained uncertain if the current availability of the vaccine, and their newly acquired knowledge of its importance, was “too little, too late” because of their age and/or HPV exposure. Improving access and uptake of HPV vaccination requires addressing both financial barriers to access as well as increasing HPV health literacy levels, particularly by reframing the long-standing gendered associations of HPV. Physicians should provide clear, tailored messages regarding HPV vaccination.

## Introduction

The human papillomavirus (HPV) is the most common sexually transmitted infection (STI) worldwide and is a cause of anogenital cancers [1]. Among gay, bisexual, and other men who have sex with men (GBM), the prevalence of high risk HPV (potentially cancer-causing HPV) in the anal canal remains high, with one Canadian study documenting these infections in 67.6% of HIV-positive and 51.7% of HIV-negative GBM participants [2]. Consequently, despite great advances in HIV treatment and care, the incidence of HPV-associated anal cancers continues to increase among HIV-positive GBM [3–4], with rates approximately 100-fold that of the general population [5–6]. Fortunately, HPV vaccination has proven efficacy in preventing both anogenital warts (condyloma) in males and anal pre-cancers (anal intraepithelial neoplasia; AIN) in GBM up to the age of 26 [7].

In 2006, the quadrivalent HPV vaccine (Gardasil) was first approved by Health Canada for females aged 9 to 26, covering the most common wart-causing HPV types (i.e., HPV-6 and -11) and the most common high risk or oncogenic types (i.e., HPV-16 and -18) [8]. The following year it was provided at no cost to girls between the ages of 9 and 13 across Canada. In 2010, approval was expanded to include males aged 9 to 26 and in 2012, Canada’s National Advisory Committee on Immunization (NACI) recommended the quadrivalent HPV vaccine for all males who have sex with males who are age nine years and older [9]. However, only in 2016 did the province of Ontario expand its coverage of HPV vaccination to include boys aged 9 to 13 years, as part of the universal school-based program, and GBM up to 26 years of age as part of a targeted program [10]. The HPV vaccine was made freely available to boys in other provinces across Canada prior to this time, and this change in policy occurred after substantive pressure from community activists, doctors, and researchers across Ontario [11].

In 2015, Health Canada approved a 9-valent vaccine (Gardasil-9), which adds five additional oncogenic types to the quadrivalent (helping to protect against anal cancer caused by HPV types 16, 18, 31, 33, 45, 52, and 58 in addition to genital warts caused by HPV types 6 and 11) [8]. However, currently only the quadrivalent vaccine is covered under provincial insurance programs. Without insurance, the out of pocket cost for all three doses of the quadrivalent HPV vaccine is around $420 CAD [12] and approximately $560 CAD for all three doses of the 9-valent Gardasil vaccine [13].

A post-hoc analysis of vaccine benefit in a cohort study of GBM aged 26 years and older demonstrated efficacy in preventing anal condylomas [14]. Studies with HIV-positive adult males [15] and boys [16] suggest that it is both immunogenic and safe in these populations, with a subsequent study in the same HIV-positive adult male population demonstrating persistent immunity over two years later [17]. One fundamental question that has remained unanswered is the utility of HPV vaccination for those who are older (i.e., >26 years) and those with known prior exposure to HPV. For example, in the United Kingdom it has been recommended that some GBM up to the age of 45 could benefit from HPV vaccination, including men living with HIV, despite previous probable HPV infection [18].

Moreover, despite the high prevalence of anal canal HPV in HIV-positive GBM, one Canadian study demonstrated that no individual had all 9 HPV types covered by the 9-valent vaccine [19], suggesting that some benefit could be derived from vaccination particularly for oncogenic strains 16 and 18. Further, a retrospective analysis of AIN recurrence rates in GBM, found that those who were vaccinated had a 50% reduction in AIN recurrence after ablative therapy (i.e. the surgical removal of anal cancer precursors or anal dysplasia) [20].

Much of the research on HPV vaccine uptake among GBM has been quantitative and has indicated that many GBM had very limited awareness of HPV’s cancer risks for GBM, but maintain a strong willingness to become vaccinated once informed of the effectiveness of the vaccine [21–24]. Some studies have also pointed to concerns with side effects [23], stigmatization [20], and vaccine effectiveness [20, 25]. HIV-positive GBM have been shown to have similar awareness and vaccine acceptability levels as HIV-negative men, and have also indicated increased levels of worry of the risks of HPV-related illness [26]. Qualitative research with GBM in North America and the United Kingdom has produced similar findings, demonstrating that GBM often report a willingness to be vaccinated despite limited awareness and understanding of HPV risks [27–28].

To the best of our knowledge, there is currently no published qualitative research that focuses exclusively on the HPV vaccination practices of GBM living with HIV in Canada. In this article, we explore HPV vaccination barriers and hesitancy among HIV-positive gay men in Canada. We conducted in-depth qualitative interviews with gay men living with HIV as part of the HPV Screening and Vaccine Evaluation (HPV-SAVE) Study. HPV-SAVE investigated HPV and anal cancer screening for gay men living in Toronto, Ottawa, and Vancouver. As part of this work, we have examined variable methods to anal cancer screening (i.e. cytology [anal Pap smears] and HPV testing) and treatment options (via a trial comparing ablative treatment to surveillance alone for anal dysplasia/pre-cancerous lesions) for HIV-positive gay men and other men who have sex with men. Participants were interviewed after undergoing anal cancer screening via cytology (i.e., anal Pap test) from their primary care doctors in Toronto. Our objective was to inductively examine participants’ narrative accounts of their knowledge, experiences, and perceptions related to HPV and the HPV vaccine in order to understand the production and organization of HPV health literacy and vaccine uptake.

## Methods

Between November 2016 and July 2017 we conducted 25 in-depth, semi-structured qualitative interviews [29] with gay men living with HIV in Toronto. 24 out of 25 study participants we interviewed identified as gay, leading us to use this language to describe our sample. However, one participant did indicate both “Two-Spirit” and “Don’t know” on their sociodemographic questionnaire (See Table 1). When quoting this participant we have specifically recognized the way in which he identified.

**Table 1:** Characteristics of interview participants.

Characteristic	n = 25	
**Age**		
Range	31 to 68	
Mean	50.44 (SD 9.99)	
**Highest Level of Education**		
Some Secondary	1	4%
Completed Secondary	3	12%
Some College	6	24%
Completed College	10	40%
Graduate Education	5	20%
**Employment Status**		
Student	1	4%
Unemployed	1	4%
Retired	4	16%
Full-Time	12	48%
Part-Time	1	4%
Other	6	24%
**Ethnicity**		
White	20	80%
First Nations	1	4%
Asian	2	8%
Other	2	8%
**Sexual Identity**	** **	** **
Gay	24	96%
Two-Spirit	1	4%
**HPV Vaccination History**		
Vaccinated before participation in HPV-SAVE	6	24%
Vaccinated after participation in HPV-SAVE	3	12%
No HPV Vaccine	19	76%

Prior to beginning the study, a community advisory board (CAB) was formed. The CAB was composed of members living with HIV, community health policy advocates, early career academics, physicians, and researchers with statistical and epidemiological expertise. The CAB met quarterly and informed the qualitative study design, interview question development, recruitment strategies, and the interpretation of preliminary findings.

Qualitative study participants were purposively recruited from a subsample of men who took part in the larger HPV-SAVE study and who indicated a willingness to be contacted for additional research activities. These participants were initially contacted by phone or email and drawn from three participating primary care clinics and one outpatient infectious disease clinic in Toronto. We recruited participants who were at various stages of the anal cancer screening process, including those who had different anal Pap results, treatment experiences, and HPV vaccination histories. We iteratively reviewed our data to inform our purposive sampling strategy following the principle of theoretical saturation [30]. See Table 1 for a summary of key participant information including HPV vaccination history and socio-demographic information.

With the exception of one interview conducted by phone for accommodation reasons, all interviews were conducted in-person at the University of Toronto in a private meeting room. Under the supervision of the first author (DG), the qualitative interviews were conducted by the second author (MG) who is a postdoctoral research fellow and a trained qualitative researcher. Participants provided informed written consent prior to the interview. Interviews were approximately an hour in length, ranging between 30–75 minutes. Participants received a $30 CAD honorarium. The study received ethics approval from the University of Toronto HIV Research Ethics Board.

The following key domains were explored: (1) general awareness and understanding of HPV and anal cancer risks; (2) social and cultural contexts informing HPV and sexual health related decision-making; (3) motivations and experiences related to anal cancer screening; (4) other anal and gastrointestinal disease and STI treatment experiences; and (5) knowledge, perceptions and experiences related to the HPV vaccine. While this last interview domain is the primary focus of the analysis presented in this paper, we draw upon the interviews in their entirety given that in most instances the HPV vaccine was discussed by participants earlier on in the interview and connected to other key interview questions, including men’s HPV knowledge and anal cancer screening motivations. Interviews were audio recorded, transcribed verbatim, and reviewed for accuracy.

QSR NVivo 11 qualitative software was used to code transcripts following Grounded Theory [30–31]. As Charmaz explains, Grounded Theory is a set of methods that “consist of systematic, yet flexible guidelines for collecting and analyzing qualitative data to construct theories ‘grounded’ in the data themselves” [32]. Using an iterative, *constant comparative method* [33], we systematically reviewed and coded transcripts as interviews were completed. We wrote codes for sections or units of texts, and created memos that expanded upon sections of coded text and described relationships across the codes. Because our qualitative coding was conducted concurrently with the data collection process, we were able to iteratively refine our interview guide and explore emergent themes in subsequent interviews [34–35].

## Results

### Vaccination history

Before enrolling in the HPV-SAVE Study, only a quarter of our participants reported being vaccinated for HPV (having received all or some of the three doses at the time of the interview). After receiving their anal Pap test through the HPV-SAVE study and discussing vaccination with their physicians, a few more participants said that they had decided to receive the HPV vaccine. Though some men stated that they made the choice to become vaccinated after receiving their first anal Pap, others described that they still felt their risk levels for HPV-related cancers remained “low” or “minimal” and they were not currently considering the vaccine.

Of our participants who were vaccinated for HPV, all reported being vaccinated as adults, usually after their HIV diagnosis. Men described no negative or unusual experiences, such as adverse events or pain, associated with receiving the vaccine. Some men expressed the positive benefits to vaccination, including that their anxieties around anal cancer risks had been substantially reduced. A number of participants noted that they had not thought seriously about vaccination until taking part in the qualitative interview. These patients said that they planned to raise the issue of HPV vaccination with their physicians during their next visit.

#### Vaccine decision-making and risk perception

Almost all of the participants we interviewed said that they were aware of the HPV vaccine before entering the HPV-SAVE study. Some men said that they first became familiar with the vaccine after they were diagnosed with anogenital warts. A history of anogenital warts increased concern over HPV-associated disease and was discussed by some participants as a facilitator to receive vaccination and anal Pap testing. At the time of the interview, only a few participants stated that they had never heard of Gardasil or the HPV vaccine before and some thought the vaccine had only been available for a few years. The majority of our participants said that they had not seriously considered or researched the HPV vaccine until after their involvement in the HPV-SAVE study. However, several participants had conversations about vaccination with their physicians in the past, but chose against vaccination.

For some participants, the decision to be vaccinated for HPV was described as quite straightforward. As one participant who had been vaccinated put it, “*If it is the sole cause of anal cancer*, *I just can't believe that people would be so stupid not to go and get [the vaccine]”* (67 years old, vaccinated). Overall, most men reported a belief that vaccines were safe, effective, and necessary. Some avidly critiqued those who were resistant to getting vaccinated: “*I'm a big vaccine person*. *I get the flu shot*, *I got the meningitis shot*, *I got the pneumonia shot*, *I do all the vaccines*. *And I don't put up with anti-vaxxers at all”* (51 years old, vaccinated).

Many participants discussed past vaccination history for other diseases/infections (e.g. flu, shingles, pneumonia) which they expressed as being a necessary part of managing their health as someone living with HIV and who is immune compromised. A few participants who were not vaccinated for HPV did note that they were interested in knowing about any potential negative side effects associated with vaccination. However, some expressed the belief that any negative side-effects of a vaccine would be offset by the benefits of preventing a serious health condition like anal cancer.

Nonetheless, despite an *emerging* sense among many participants that HPV and anal cancer are serious health risks to HIV-positive men, and a reported interest by many to be vaccinated, most men in our sample had not received the vaccine and many remained ambivalent about HPV vaccination. In the sections that follow, we present a review of the two overarching and multi-layered factors affecting the vaccine hesitancy of our participants: (1) *the association of HPV with women and low levels of vaccination literacy* and (2) *the role of vaccine price including persistent financial barriers to vaccine access*. We argue that both of these interrelated factors are a product of public health policy and health promotion strategies that have governed HPV vaccination in Ontario, Canada.

### HPV, gendered risk perceptions, and vaccination knowledge

Many participants discussed having first heard about HPV and the HPV vaccine in the media. However, almost all participants reported initially believing HPV vaccination was predominately or exclusively an intervention designed for cisgender girls or women and it was for the prevention of cervical cancer. For many, this belief persisted until enrollment in the HPV-SAVE study. One participant who was not vaccinated explained: “*I've always associated it to cervical cancer and to women*. *I didn't realize at the time*, *until recently*, *that it affected men as well*…” (43 years old, not vaccinated). This man went on to explain how his knowledge of HPV was shaped:

*I had heard of it [HPV vaccine]*. *Again*, *it was in those commercials*. *But again*, *my own ignorance was that HPV was something that really only affected women and cervical cancer*. *I didn’t hear it as much as something that was affecting men*. (43 years old, not vaccinated)

Participants’ earliest reported recollections of the virus tended to revolve around education and vaccine promotion campaigns addressing women about the risk of cervical cancer. Another participant, who recently became vaccinated, reflected on his first exposure to messages about HPV vaccination in similar terms: “*…it seemed to be young*, *teenage girls was the context that I had heard about it*, *and it wasn’t ‘til recently that I heard that it was affecting men as well*, *right*? (59 years old, vaccinated, Two-Spirited).

The perceived association between HPV and women appeared to be a significant factor affecting the vaccine hesitancy of the participants we interviewed. Of those participants who reported that they were aware that HPV affected GBM prior to enrollment in the HPV-SAVE study, many still considered cervical cancer to be the predominant health outcome of an HPV infection, and only recently learned that anal cancer was a potential risk to them. Some participants also noted that they had not considered anal cancer as part of the male-specific cancers that they were knowledgeable of, such as prostate, testicular, and even colon cancer.

Many participants reported low levels of health literacy regarding HPV risks among GBM before entering the HPV-SAVE study, stating that they had not considered HPV a serious health concern and, as such, never initiated a conversation about HPV with their physician. Some participants described that the association between HPV and women made it hard for them to locate health promotion material that was directly relevant for them once they realized that they could be at risk:

*Then I heard—and I thought*, *oh my God*, *something that women have to deal with that I just don't have to deal with*. *That's what I thought*. *And [I] tried to look up HPV and there wasn't a whole lot for men*. *It was…so I thought*, *okay*, *well*, *it doesn't seem to be an issue [laughter] in men*. (30 years old, not vaccinated)

In another related account, one participant discussed encountering HPV-related information in a health booklet he received soon after his HIV diagnosis. This man reported that he did not think about HPV or vaccination at the time because he thought it was predominantly a women’s issue and there was so much new information he was trying to process after his HIV diagnosis:

*More women*, *I mean*, *I read in [pause] when you first get diagnosed [with HIV]*, *you have this huge book they give you*. *So I think I sort of [had] gone through it*, *but again*, *you're sort of all over the place at the time*, *so I didn't really focus on anything*. *And in general*, *I'd thought the HPV was…I'd heard about vaccines for girls and stuff like that and there was some talk about boys getting it*, *but I had never really thought about it*, *to be honest*. (47 years old, not vaccinated)

#### Gendered associations and the role of physicians in decision-making

Once informed by their primary care physicians and/or HPV-SAVE researchers about the risks posed by HPV to gay men in the form of anal cancer, most men explained that they could understand how this could be the case and were interested in learning more and becoming vaccinated. In other words, in our sample, the gendered associations held about HPV appear to have impacted HPV risk perception and vaccine health literacy levels going into our study. However, these gendered associations did not appear to lead to a resistant or refusing attitude toward accepting HPV as a relevant health concern once the risks were clearly presented to participants.

However, some of our participants reported that their physicians had never brought up either HPV or the HPV vaccine to them. Others expressed that they may have encountered the topic of HPV and vaccination in a clinical setting before, but the details and the stated importance of vaccination remained somewhat vague. None of our participants reported that the vaccine was *strongly* recommended by a physician and then they decided to not be vaccinated—the vaccine may have been recommended but it was still presented as *optional* from the perspective of participants.

Finally, some men’s decision to not be vaccinated appeared to be rooted in uncertainty over whether or not it remained useful to receive the vaccine now, given that they most likely had already been infected with HPV: “*I'm under the impression also that it's a little too…too little*, *too late for me [laughs] for the vaccine*, *because I mean*, *I already have it [HPV]*” (37 years old, not vaccinated). Many patients described requiring a very clear recommendation about the vaccine from their physician before they would get the vaccine. In the absence of this recommendation, they said that they would not actively pursue HPV vaccination.

### Cost as a complex barrier to vaccine access

For the sample of participants who were vaccinated, most described that they relied on private insurance to cover the cost of the vaccine. For example, one participant talked about how vaccination was “*easy*” as his insurance “…*covered it completely*. *I just had to pay and submit the receipts”* (52 years old, vaccinated). When asked if his decision-making would have changed if he had to pay out of pocket for all or a portion of the HPV vaccine, he went on to explain:

*Wouldn't have made any difference*. *I would've gone ahead with it*. *And I remember saying at the time because [my doctor] said to me*, *the vaccines*, *both of them—there was a third one I had*, *I think the third one I had was pneumonia or something—anyway*, *in any event*, *he said you know*, *they may be expensive*, *you may not be covered*, *do you want to check first*? *I said no*, *I'm getting it done anyway*. *You know*? *I'm getting it done*. *That's a minor investment*, *whatever it is*, *towards your health*. (52 years old, vaccinated)

The above participant described thinking about the HPV vaccine as he did with other vaccines that were recommended by his doctor: they were necessary and important for his health regardless of expense. Another man, who had insurance to offset the vaccine cost, explained it this way when asked if cost was currently a barrier in his decision-making to get vaccinated: “*Well*, *no*. *Not now that I have more information about it*. *I think it’s one of those things that I would do just because it’s the safe thing to do*” (43 years old, not vaccinated).

These accounts can be read alongside the narrative of another participant who was vaccinated *after* he received abnormal tests results from his HPV-SAVE anal cancer screening. He described that his vaccination costs were covered by insurance. However, if this were not the case, he said he would be uncertain about whether the benefits of vaccination—given that he had already been infected with HPV—justify the significant out of pocket expense.

*After being diagnosed [with anal dysplasia]*, *not knowing if [the vaccine is] going to have any benefit…I don’t know*. *It would depend on the cost*. *If it’s a high cost*, *then probably not*. (39 years old, vaccinated)

Some of our participants articulated that cost was the *only* factor leading them not to become vaccinated. For example, one participant stated that his physician prescribed the HPV vaccine to him; however, he ultimately did not end up filling his prescription for the vaccine because of the prohibitive price. Another patient made it very clear that he both perceived himself to be at a significant risk of HPV related cancers and that he and his doctor decided that vaccination was the best course of action. Despite a very strong desire to be vaccinated, he decided against vaccination because he was simply unable to afford it, which he expressed with great frustration.

Even when physicians were said to have made unambiguously clear recommendations for HPV vaccination, the perceived benefits and necessity of the vaccine were weighed against significant affordability concerns by some participants. Only a few unvaccinated participants thought that they could use their private insurance to cover all or a majority of the costs. Others stated that they were willing to pay completely out of pocket, despite the financial challenges, given the health benefits of vaccination as they now understood them. As one participant explained, even if he did not have the money right now—and “can’t afford it”—he would find a way to pay for the vaccination:

*The less [cost] possible*, *but [laughs] at the same time*, *I'm going to pay—oh*, *I think she mentioned that it was expensive*. *I don't—is it $200*? *I don't know*. *But I think she mentioned something*, *that it was expensive*, *and I thought oh*, *damn*. *But it's health*. *So if I can’t afford it*, *I will afford it*. (43 years old, not vaccinated)

One man who reported considering vaccination, stated that he would overcome the cost barriers by delaying the vaccination process: “*It would [be] a hit for me*, *but if I knew it in advance and maybe I'd have to wait a little bit longer to get it*, *but I would definitely do it* (47 years old, not vaccinated). Of those who reported being open to vaccination, others stated that they were only willing or able to pay a portion of the cost, often citing between $35 to $100 CDN as a reasonable amount to pay out of pocket. One man put it like this:

P: *Well*, *you know*, *if you said it was $500*,*000*, *no*. *If you said if was $100*, *perhaps [laughs]*.

I: *How about $500*?

P: *No*, *I wouldn’t*. (59 years old, not vaccinated)

When responding to questions about the willingness to pay for the HPV vaccine and weighing the costs and benefits of vaccination, some participants explained not knowing how many shots would be needed of the HPV vaccine: “*It’s going to be like long-term*, *or you have to get [vaccinated] like every certain time*?*”* (46 years old, not vaccinated). Other participants thought about cost in relation to frequency of other vaccines, most notably the flu vaccine:

P: *I've never paid $350 for a vaccine*.

I: *So that would be too high*?

P: *That would be*… *you know*, *once again*, *how often*? *Like the flu shot you have to get every year*, *but I don't know the HPV*, *I don't know the frequency or anything*. *I don't know that*. (30 years old, not vaccinated)

## Cost and perspectives on policy

Only a few participants reported being aware that the HPV vaccine is freely available to men in Ontario who are 26 years old and under. When informed about coverage, most said they saw this as important even if they were not able to benefit personally from this recent shift in coverage. Some men, however, did express discontentment over previous and current strategies governing HPV vaccination coverage. For example, one participant argued that the previous approach focusing only on cisgender girls and women was an example of the government being “*sexist*” and described the issue of HPV among gay men with a high degree of urgency: “*Like*, *this is not something we should be playing around with*. *This is something that should've been done a long time ago*, *because too many people’s lives depend on it now* (31 years old, vaccinated).

Another participant described having a very strong interest in being vaccinated but was prohibited entirely by the costs. He vocalized very strong critiques against the current health policies restricting free access to gay men and other men who have sex with men:

*I think the government's being short-sighted*, *that covering things like this is*, *in the long run*, *a far smarter thing to do*. *You pay a couple of hundred bucks for a vaccine or you pay thousands of dollars for someone's chemotherapy treatment*. *It's just*, *the math is ridiculous on these things*. (53 years old, not vaccinated)

When informed about coverage for boys and men, including younger gay men, he also vocalized frustration: “*Yeah*, *but again*, *why under 26*? *I mean*, *that's ridiculous*. *It's… very frustrating*.*”*

## Discussion

Our interviews were an opportunity to qualitatively investigate HPV vaccine decision-making among older HIV-positive gay men in Toronto, Ontario, Canada. Among the participants we interviewed, men reported no significant degrees of resistance to the general idea of being vaccinated for HPV. The majority of participants did not express significant concerns about side effects, vaccine efficacy, or the necessity of being vaccinated. When presented with the evidence on HPV-associated cancer risk for GBM, most participants became interested in vaccination. Despite this, the majority of the men in our sample have not been vaccinated for HPV, and many remained undecided about vaccination, even after they had been screened for anal cancer as part of their engagement in the HPV-SAVE Study.

Two primary factors appeared to have facilitated a degree of vaccine hesitancy or reluctance among our participants. The first was a primary lack of risk perception and health literacy about HPV cancer risks for men, including gay men. The association between HPV, women, and cervical cancer played a significant role in shaping our participants’ interpretation of themselves as being at risk for HPV and the limited information they had about HPV and anal cancer. Even when participants were aware of HPV risks, for many, these gendered associations made HPV less of a priority. Some participants remained hesitant about the HPV vaccine even after they had received abnormal cytology and histology test results and were undergoing treatment for anal dysplasia. All participants who articulated that HPV vaccination was not *strongly* recommended or encouraged for them by their primary health care provider said that they decided not to be vaccinated. Our research supports the findings of previous studies that have found physicians’ recommendations to be highly correlated with an increased likelihood of patients accepting to be vaccinated for HPV [24], underscoring the importance of receiving clear recommendations from primary health care providers.

The second primary factor that led to vaccine hesitancy or reluctance was the price/out of pocket expense of the vaccine. Participants’ decision-making processes regarding vaccination appeared to be frequently shaped by cost. In men’s narrative accounts, risk perception and cost appeared to inform men’s decisions both independently and co-constitutively. Sometimes participants reported not pursuing active questioning about the HPV vaccine with their physician, even when they had the resources and insurance to pay for it, as they did not consider themselves to be at high risk. Other participants expressed concerns about the significant costs of the vaccine, but their strong sense of individual risk perception appeared to make them decide that vaccination was the right health decision for them. However, some patients who considered themselves to be at a significant risk and the vaccine to be a valuable intervention, remained reluctant or hesitant due to the cost. Finally, some participants explained that they understood themselves to be at risk of HPV-related illnesses but they were unclear if they would benefit from vaccination, having likely been infected with HPV already. This is despite the fact that the NACI recommends vaccination for all GBM over the age of 9 [9].

As such, notwithstanding their noted acceptability of vaccination (i.e., the idea/principle of getting vaccinated in general) reluctance remained in relation to HPV vaccination. Viewed in this way, our participants appeared to be closer to the “acceptance” end of the vaccine hesitancy continuum [36], with a number of interrelated obstacles preventing vaccination. Our analysis points to a variety of interrelated *structurally produced* vaccine hesitancies. Rather than a property of individuals refusing vaccination, conceptualizing the production of vaccine hesitancy in this way elucidates how social systems—i.e. media, health promotion campaigns, public policy, physician recommendations—serve to create environments and knowledge systems in which people are less likely to become vaccinated due to dominant discursive frames of risk and persistent resource barriers to biomedical interventions.

Given these structurally produced hesitancies in our sample, it was evident that a clear and strong recommendation by a physician was a primary facilitator to be vaccinated. This poses challenges for physicians who have to balance communicating the possible benefits of vaccination with respecting how their patients’ decision-making may be constrained by finances. However, physicians should be mindful not to communicate the vaccine as being less essential because of the costs. Physicians serving HIV-positive patients should readdress the issue of vaccination annually, to see if a patient’s financial situation has changed, and to clarify the benefits of vaccination.

This qualitative analysis complements emergent quantitative findings on HPV vaccination with HIV-positive men in Ontario [37]. For example, among 678 men interviewed using a structured questionnaire, many had either not heard of HPV (20%) or had heard the term but did not know what it was (25%). Among men familiar with the term (n = 398), only 51% knew that HPV can cause anal cancer and 56% knew that people with HIV are at higher risk for cancers caused by HPV. Many thought their chance of getting HPV was zero (19%) or low (36%). Sixty-three percent had heard of the HPV vaccine and 44% knew that it was recommended for males, but only 13% reported that a health professional discussed the vaccine with them and 6% were vaccinated. Men said that they would be likely/very likely to get vaccinated if it were offered free of charge (81%), if they had to co-pay $30/dose (59%), or pay full price (18%).

Our work also complements international epidemiological literature that has found limited awareness of HPV vaccine availability for men [17, 25], the belief that HPV is predominately a risk for cervical cancer among cisgender women [38], and a limited understanding of anal cancer risks [17–18, 20], as persistent barriers to vaccination. This quantitative research has also underscored that the costs of the HPV vaccine are major impediments to vaccine uptake [17–18, 24, 39].

Our analysis is subject to several limitations. We exclusively focused on HIV-positive GBM. These men may have different understandings of the necessity of the HPV vaccine than HIV-negative GBM, because they are at higher risk for HPV associated cancers [31]. Our participants also tended to be older (mean age of 50.4 years old), white, and identified as gay males. We did not interview any transgender males. Our participants were also recruited from a clinical trial on anal Pap testing and thus the participants may be more active in their health care than the general GBM population. Nonetheless, that we found vaccine hesitancy and low awareness of the HPV vaccine among a group of highly active health seekers, may indicate just how significant the problem may be across a more generalized population of GBM.

## Conclusion

Public health policy and health promotion strategies in Canada have facilitated vaccine hesitancy among HIV-positive men at risk for HPV associated cancers. In Canada, the lack of coverage for HPV vaccination among HIV-positive men of all age groups, and health promotion strategies and media having focused on cisgender girls and women, have played a role in producing vaccine hesitancy among men who are at significant risk for anal cancer–especially GBM living with HIV. Fortunately, current health promotion and media efforts are focusing more on boys and men, and several Canadian provinces currently have “high-risk” HPV vaccination programs for males [40].

Improving access and uptake of HPV vaccination requires addressing both persistent financial barriers to access as well as increasing HPV health literacy levels among men *and* healthcare providers [41] particularly by reframing the long-standing gendered associations of HPV and emphasizing vaccination as part of a holistic safer sex strategy. The feminization of HPV and HPV vaccine messages shaped the knowledge base of our participants, leading many to think that the HPV vaccine was not relevant for them. Men’s accounts of the gendered messages of HPV demonstrate that once an association has been strongly communicated about a particular health concern, people may interpret and process new knowledge through this established frame. Even though policies and messaging of a health risk may change over time, the initial gendered associations communicated can play a strong role in shaping how individuals process new health messages and programs. However, while the feminization of HPV related cancers has undoubtedly played a role in these men’s risk perceptions of HPV and the importance of HPV vaccination, it did not appear to serve as a permanent or insurmountable barrier to men’s health decision-making. Increasing HPV vaccination among HIV-positive GBM in Ontario and across Canada will require the commitment of well-informed health care providers who provide clear recommendations along with continued public health, governmental, and community efforts to remove barriers to access.

## Supporting information

S1 File Interview guide. (DOCX)
