# Supplementary material for: HIV-positive gay men’s knowledge and perceptions of Human Papillomavirus (HPV) and HPV vaccination: A qualitative study
Source: PLoS One. 2018 Nov 29;13(11):e0207953. doi: 10.1371/journal.pone.0207953 (PMC6264470; doi:10.1371/journal.pone.0207953)
Supplement: S1 File — (DOCX) [file pone.0207953.s001.docx]

**HPV-SAVE Qualitative Interviews**

***Interviews with Patients***

**[Informed consent process completed]**

Thank you for agreeing to take part in this interview.

A key aim of the HPV-SAVE Team Grant is to gain an in-depth understanding of how anal cancer screening, treatment and vaccine programs can best be delivered to HIV-positive gay, bisexual and other men who have sex with men.

As part of this research we are conducting qualitative individual interviews to learn from both patients and physicians across a number of Canadian cities.

**General Awareness of HPV Risk**

1. Do you remember when you first heard of the human papillomavirus or “HPV”? Could you tell me about that?
2. Do you feel knowledgeable about HPV? Why is that?
   1. Could you tell me what HPV is in your understanding? Do you know how HPV is transmitted?
   2. Is HPV preventable? How? Is HPV curable? How? How effective are condoms at preventing HPV?
   3. Are you aware of the relationship of HPV with other diseases, including anal cancer? Including anal warts? Genital warts? To other sexually transmitted infections (STIs)?
   4. What is the relationship between HPV and HIV? Probe: Have you heard that having HPV can make the transmission of HIV easier?
   5. Do you know about the oral transmission of HPV? Is this a concern to you?
   6. Have you heard of penile cancer related to HPV? Is this a concern to you?
   7. Do you know of any risk factors or risk behaviours that increase the likelihood of someone getting HPV? Or that increase the likelihood of someone getting anal cancer? (E.g. gay, bisexual and other men who have sex with men, HIV status, smoking)
   8. Do you know that there are different strains of HPV? What are your thoughts on this?
   9. Do you know the difference between anal cancer screening and colon cancer screening (colonoscopy)?
   10. Is there something that you feel you don’t know about HPV that you want to learn more about? Why is that?
   11. Could you tell me about how you learned about HPV? Probe: Doctors, personal experience, friends, informational brochures, websites etc. Where would you like to acquire further information in the future?
3. I am interested in learning if a healthcare provider has ever told you that you had:
   1. Human Papillomavirus or HPV?
   2. Genital or anal warts?
   3. Anal cancer or anal pre-cancer [anal intraepithelial lesions (AIN) or anal dysplasia]?
4. *Modify questions based on the answers above.* Could you tell me what you think your chances are of getting the following:
5. Human papillomavirus or HPV in your lifetime? Why do you think that?
6. Genital or anal warts in your lifetime? Why do you think that?
7. Anal cancer in your lifetime? Why do you think that?
8. When thinking about your health (physical, mental, sexual)—living with HIV, the treatments you are taking, your medical appointments, other infections or issues—how does HPV risk and anal cancer fit into all of this for you?
9. How important is HPV as a health concern for you? Why is that? Is HPV more or less of a concern as other STIs to you?
10. How important is anal cancer as a health concern for you? Why is that?
11. Is there anything important or useful that you’ve learned about living with HIV that you think might be helpful in how we think about HPV and anal cancer?
12. What do you think are some of the ways in which HIV-positive men can reduce their risks of HPV infection and anal cancer?

**HPV, Sexual Practice and Social Context**

1. How important do you think anal cancer or HPV is as a health concern for gay men in general? For gay men living with HIV? Why do you think that is? Do you perceive there to be a difference between these groups?
2. Do you think there are generational differences when thinking about HPV?
3. Have you talked with any of your friends (straight, gay, HIV-positive, HIV-negative) about HPV, anal cancer screening or treatment experiences? Are there differences of opinion? Do you think people are talking enough about HPV? Why do you believe this?
4. Do you think the association between HPV and cervical cancer has affected how *men* think about the disease?
5. Do you think that because we are talking about the anus this affects how people talk about anal cancer and HPV? Why is that the case?
6. *Modify based on experiences/treatments discussed.* I am interested in understanding how any experiences you have had related to HPV and anal cancer screening and treatment may have impacted your sex life (including safer sex decisions)?
7. Do you think people need to adjust their sexual practices (their sexual behaviours or safer sex choices) to factor in HPV and anal cancer more? If so, how?
8. Do you think that people need to disclose to their new sexual partners if they have (or have had) HPV? If they think that they might have HPV? If they have (or have had) anal pre-cancer or cancer? Is this your same opinion for other STIs?
9. How do you think recent advancements in HIV prevention will affect HPV and anal cancer risk? (Probe: PrEP, probe undetectable viral load).
10. Are there any other social issues (explain: how people are interacting with each other) that you think I should be aware of related to HPV and anal cancer?

**Anal Cancer Screening and Health Care Experiences**

1. Did you receive an invitation for Pap testing?
2. If yes, did you have your Pap test already?
3. I would like to ask about the invitation for Pap testing you received. We are interested in learning about why you were (or were not) motivated to be tested (for anal cancer) and the role different kinds of invitations for screening may have played.
   1. Did you receive a letter from your doctor for a Pap test? Another means of invitation? Did this inform your decision to get tested or not to get tested? *Probe (if received letter)*: Is there anything that could have been added to the letter that would have been helpful?
   2. Have you ever had any anal cancer screening procedures *before* the invitation for this Pap testing?
   3. Had you considered going in for Pap test or anal cancer screening *before* you got this letter?
   4. How knowledgeable do you feel about anal cancer screening? For example, could you tell me what it is? What procedures are involved? Who does it? Does everyone get it as part of their routine care?
   5. Were you clear on what the Pap test was *before* going into the exam?
   6. Did you feel prepared going into the exam? Was there anything else you wish you knew before the exam? Any surprises during the exam?
   7. Could you explain your experience (what happened during the screening process)?
   8. Do you know what the doctor was looking for? Did you feel that you had enough information about the exam?
   9. Were you comfortable during your experience? Was there anything that could have made you more comfortable?
   10. Did you receive your results from the pap test? Do you have any comments about this process? Were you concerned about what your results might be?
4. How comfortable are you with discussing health issues related to your sexual health with your HIV doctor? How comfortable are you discussing issues related to your anus with your doctor? Why do think this is the case (why are you uncomfortable or uncomfortable)? If uncomfortable: what could make you feel more comfortable about talking about your sexual health and anus? If comfortable: was this always the case? If not, how did you develop this comfort?
5. Could you tell me about any other experiences you have had where an anal swab was performed by your doctor looking for *sexually transmitted infections* (not just for anal cancer)? Do you have any comments on this procedure? When was the most recent time it was performed? Why was this preformed?
6. Have you ever had a digital anal rectal examination where a doctor inserted his or her finger inside your anus? Or a high-resolution anoscopy exam? This is where you would have gone to a specialist and a scope and a special microscope was used to do an internal exam and biopsies may have been taken. Do you have any comments on this procedure? Why was this preformed?
7. Have you ever had a colonoscopy?
8. Have you ever had treatment for lesions caused by HPV?
   1. Probe: Did you have surgery, use creams, cryotherapy/freezing, burning, acid, or ablative therapy (i.e. infrared coagulation or electrocautery)? I am interested in your experiences related to this treatment.
   2. Probe: Instead of treatment, have you been monitored (e.g., watchful waiting)? I am interested in your experiences related to this monitoring.
9. Have you ever had treatment for HPV-related pre-cancer or anal warts?
   1. If yes, could you please tell me about your experience? Probe: How was the treatment process? Was it painful? Were you nervous or anxious?
   2. Did you have to wait long for your appointment? Would you have liked your appointment to be earlier?
10. Have you had any other healthcare experiences related to anal cancer screening I may have missed?
11. Thinking about these experiences in the clinic, do you have any comments on your overall health care experience? For example, other people you interacted with besides the doctor? Do you have any suggestions for improvement?
12. Building on your experiences to date, would you be interested in anal cancer screening as part of your regular health care routine? Why or why not? What would make you want/not want it? What do you see as barriers?
13. How often do you think you should be screened for anal cancer? How often are you willing to go?

1. Did (or would) the recommendation to have anal cancer screening from your doctor inform your decision-making process?

**Vaccine**

1. Before being apart of this study, had you ever heard of Gardasil/the HPV vaccine?
   1. Have you ever received the HPV vaccine? *If yes:* If so, how many doses? Who paid? How old were you when you received your first dose of the vaccine? Do you have any comments on your experiences of receiving this vaccine?
   2. *If no:* Has your healthcare provider ever discussed this with you? Has it ever been specifically recommended/not recommended for you? Would you consider getting it? If so, would you pay? How much would you be willing to pay?
   3. Who should get this vaccine? Should it be available to everyone?
   4. What do you see as barriers to accessing this vaccine? Probe: would having to pay for the vaccine be a barrier for you?
   5. Do you have concerns about the HPV vaccine? (Probe: safety, effectiveness, etc.)
   6. Do you usually get vaccines? Are you against them?
   7. Are you aware that gay men/MSM under 26 in Ontario can now get the cost of HPV vaccine covered? How do you feel about this? Do you understand why the policy focuses on people younger than 26? What do you think about men over the age of 26 (HIV-negative and HIV-positive), do they need coverage?
   8. Did you know that girls and women had the vaccine covered before boys and men got coverage? How do you feel about this?
   9. Do any of these policy issues –that men and boys were originally not covered—affect how you think about the HPV vaccine? Anal cancer?

**General Overview and Conclusion**

1. Finally, do you have any additional comments to share regarding how anal cancer screening, treatment and vaccine programs can best be delivered to HIV-positive gay, bisexual and other men who have sex with men?
2. Thinking about our discussion what information and what services, if any, do you feel that you need? What recommendations do you have for us on HPV education?
3. Do you have any feedback about this interview?

**Thank you for your participation in this interview.**
